# Miscellaneous Medical Intelligence

**Published:** 1851-09

**Authors:** 


### ARTICLE V.

### MISCELLANEOUS MEDICAL INTELLIGENCE.

*Health of Chicago.*—The Cholera has been prevailing to a limited extent in Chicago since early in July, the deaths ranging from one to six a day from that time to the present. So far the season has been much more healthy than for several years past. The Dysintery, though attracting less public attention than Cholera, is much more prevalent, yet it is generally mild in its character. A few cases of Remitting fever have presendte within the last week or ten days.

*New Journal.*—We have received the Nashville Medical Journal, and welcome it to our list of exchanges. It is well got up, and filled with interesting matter. We wish it success.

The New Jersey Medical Reporter has been changed from a quarterly to a monthly visitant, and is always welcome.

The Transactions of the College of Physicians are now published quarterly at one dollar per annum. It is one of the most interesting and useful periodicals of the country.

The forthcoming volume of the Transactions of the American Medical Association will contain the prize Essay on Ovulation of Prof. J. C. Dalton, jr., of the Buffalo University, embellished with several fine engravings. Members of the Association can obtain it by remitting two dollars to I. Hays, M. D., Treasurer, Philadelphia.

By the language of a notice of Prof. Armor's appointment to a chair in the Cleveland University, in a Cleveland paper marked and sent to us by some one, in which he is styled late Professor in the Iowa University, we were led to infer that he had resigned his place in the latter School; but we observe that instead of this, he has not only accepted the Chair in the Cleveland College referred to, but has had an additional Chair assigned to him in the Iowa School—being now Professor of Physiology, Pathology and Clinical Medicine, and also of Theory and Practice.

We have the pleasure of presenting our readers with another number of our Journal entirely filled with original matter.

